# Are Fungal Endophytes Merely Mycorrhizal Copycats? The Role of Fungal Endophytes in the Adaptation of Plants to Metal Toxicity

**DOI:** 10.3389/fmicb.2019.00371

**Published:** 2019-03-15

**Authors:** Agnieszka Małgorzata Domka, Piotr Rozpaądek, Katarzyna Turnau

**Affiliations:** ^1^ Institute of Environmental Sciences, Jagiellonian University, Kraków, Poland; ^2^ Malopolska Centre of Biotechnology, Jagiellonian University, Kraków, Poland

**Keywords:** fungal endophytes, toxic metals, mycorrhiza, phytoremediation, phytomining

## Abstract

The contamination of soil with toxic metals is a worldwide problem, resulting in the disruption of plant vegetation and subsequent crop production. Thus, remediation techniques for contaminated soil and water remain a constant interest of researchers. Phytoremediation, which utilizes plants to remove or stabilize contaminants, is perceived to be a promising strategy. However, phytoremediation’s use to date is limited because of constraints associated with such factors as slow plant growth rates or metal toxicity. Microbial-assisted phytoremediation serves as an alternative solution, since the impact of the microbial symbionts on plant growth and stress tolerance has frequently been described. Endophytic fungi occur in almost every plant in the natural environment and contribute to plant growth and tolerance to environmental stress conditions. Although this group of symbiotic fungi was found to form association with a wide range of hosts, including the non-mycorrhizal Brassicaceae metallophytes, their role in the response of plants to metal toxicity has not been thoroughly elucidated to date. This review summarizes the current knowledge regarding the role of endophytic fungi in the tolerance of plants to toxic metals and highlights the similarities and differences between this group of symbiotic fungi and mycorrhizal associations in terms of the survival of the plant during heavy metal stress.

## Introduction

The deposition of toxic metals (TMs) in the topsoils of a significant acreage of land has become a major problem over a wide range of countries from both highly developed and developing regions of the world. Increasing quantities of Zn, Cd, Pb, and Fe that are produced by sewage discharges, mining operations, and runoff from metal-refining industries have severely limited vegetation what negatively affects numerous branches of human activity including food production/agriculture, urbanization, tourism, and other human practices. Difficulties in restoration of metal polluted environments arise from: (1) metal toxicity, (2) low nutrient content, (3) poor physical structure of the substrate, and (4) degraded microbial communities (Rajkumar et al., 2009).

In the natural environment, plants interact with a multitude of symbiotic microorganisms. This interaction includes mycorrhizal fungi (ecto- and endomycorrhizae), other rhizosphere-borne plant growth promoting microorganisms (PGPMs), such as rhizobial and endophytic bacteria, including plant growth promoting rhizobacteria (PGPR) and endophytic fungi (Vandenkoornhuyse et al., 2015). The role of beneficial fungal symbionts in plant metal accumulation and tolerance has been underestimated for many years; however, it has attracted more interest recently (Rajkumar et al., 2009, 2012; Weyens et al., 2009b; Burges et al., 2017). Significant progress has been made in understanding the role of mycorrhizae in metal stress tolerance, and several comprehensive reviews have recently been published (Meier et al., 2012; Cabral et al., 2015; Ferrol et al., 2016; Coninx et al., 2017; Das et al., 2017; Miransari, 2017; Rozpądek et al., 2017). Besides mycorrhiza, another group of symbiotic fungi – fungal endophytes – has been attracting the interest of the scientific community due to their potential beneficial impact on vegetation, including in the facilitation of plant growth metal polluted environments. Endophytic fungi are a taxonomically diverse group of ubiquitous cryptic microorganisms that reside inside their host without causing any visible symptoms of infection for at least part of their life cycle (Rodriguez et al., 2009; Purahong and Hyde, 2011; Sridhar, 2012). Based on the host range, *in planta* colonization, mode of the transmission and biodiversity, two primary groups (divided into 4 classes) of endophytic fungi have been distinguished. The group of clavicipitaceous (C) endophytes (class 1) is closely related species, colonizing systemically cool- and warm-season grasses (Bischoff and White, 2005). While, among non-clavicipitaceous (NC) endophytes, three broad-host range classes can be distinguished, including fungi colonizing systemically host tissues (class 2), fungi growing exclusively in plant above-ground tissues (class 3), and those restricted to plant roots (class 4) that include dark septate endophytes (DSEs) belonging to Ascomycota and non-mycorrhizal members of Sebacinales, Basidiomycota (Rodriguez et al., 2009; Andrade-Linares and Franken, 2013).

**Figure 1:**
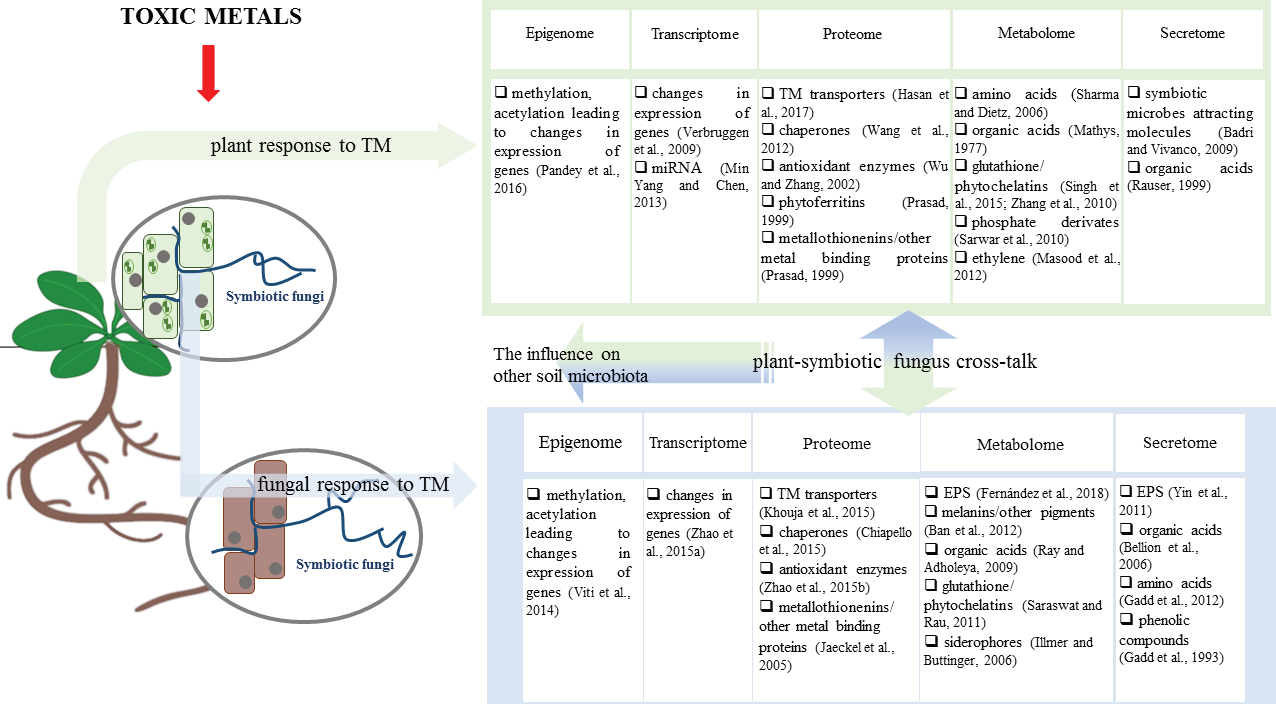
Influence of toxic metals (TMs) on the plant, symbiotic fungi, and their symbiosis. Metal toxicity exerts a substantial influence on plants and fungi leading to changes in the responses in the epigenome, transcriptome, proteome, metabolome, and secretome. Therefore, the interactions between the plant and symbiotic fungi (and also other microorganisms) may be affected through the effect on factors, such as the composition of the plant and fungal secretome. The resulting symbiotic associations subsequently affect both symbiotic partners leading to the enhanced response to metal toxicity.

Fungal endophytes positively affect vegetation as follows: (1) indirectly by soil formation, which is particularly important in degraded environments and (2) directly by fine tuning the adaptation of the plant to metal toxicity and improving the plant biomass yield (Figure 1) (Li et al., 2012b; Coninx et al., 2017; Rozpądek et al., 2018). These traits of fungal endophytes make them attractive for plant-based environment restoration technologies (phytoremediation).

In this review, we summarized the studies available on the role of endophytic fungi in conferring plant toxic metal tolerance, with a particular emphasis on NC endophytes. We also attempted to address the issue of the differences between this group of symbionts and other groups of fungal mutualists, particularly mycorrhizae, in the context of fungal-dependent mechanisms that enable the plant-microbe consortia to survive under the challenge of toxic metals.

## Endophytic Fungi: Lifestyle and Taxonomy

Endophytic fungi exhibit a high degree of lifestyle versatility, and depending on the genetic traits of the partners, developmental stage, nutritional status, and other environmental factors, they can interact with their host in mutualistic, commensalic, or as latent pathogenic as summarized by Schulz et al. (2002). Such versatility and the loss of virulence can be the result of a single mutation or an epigenetic change due to changes in the environment. However, plants colonized by the endophytic fungi show improved growth, better performance, induced resistance, and the biocontrol of nematodes, fungi, and pathogenic bacteria and fungi. The asymptotic colonization of the plant relies on the balance of the antagonism between the host and the endophyte. This balance can be destroyed when the environmental factors change or the plant reaches the stage of senescence (Deckert et al., 2001).

NC endophytes include Ascomycota, Basidiomycota, and Mucoromycotina (Huang et al., 2001; Rosa et al., 2010; Andrade-Linares and Franken, 2013) that were shown to be able to inhabit bryophytes, ferns, gymnosperms, and angiosperms (trees, shrubs, and herbs), including the non-mycorrhizal Brassicaceae, particularly abundant in metallophytes and hyper-accumulators. The association of Mucoromycotina with ancient bryophytes and liverworts is thought to represent an ancestral plant-fungal interaction and probably played a crucial role in the terrestrialization of plants (Bidartondo et al., 2011). These fungi could have developed from saprophytes that first adapted to become endophytes, and later, those inhabiting the below-ground parts of the plants could have developed into mycorrhizal fungi (discussed in Strullu-Derrien et al., 2018). The interaction certainly took place several times during evolution; thus, the endophytes evolved within distant phylogenetic groups of fungi in a manner similar to that of the mycorrhizal fungi, which facilitated the interaction with almost all plants. The beneficial interaction of the host and its microbiome, including bacteria, archea, and fungi, is responsible for maintaining the health of the plant (Syamala and Sivaji, 2017).

Endophytic fungi are facultative plant symbionts (facultative biotrophs) and according to Brundrett (2002), in contrast to mycorrhizal fungi, their development is not synchronized with the development of their hosts. Thus, fungal endophytes may complete their lifecycle outside the host organism and thus are able to grow on artificial media (Petrini, 1996), which facilitate the manufacture of pure inoculum under sterile conditions and eliminate the difficulty of their propagation. This fact is particularly important in relation to phytoremediation and the large-scale production of inoculum.

Another distinct feature of the fungal endophytes (species belonging to classes 1, 2, and 3) that distinguishes them from mycorrhizae is their ability to colonize the above-ground organs of the plant. In contrast to mycorrhizal fungi, NC endophytic fungi were found in plant leaves, stems, flowers, and seeds (Yuan et al., 2010; Hardoim et al., 2015), while mycorrhizae are restricted to the roots of plants. In addition, a large number of species can exist in the form of mycelia or form yeast-like structures or mycosomes (Atsatt and Whiteside, 2014). This aspect of endophytic fungal biology is particularly interesting because the morphological changes described may occur during the colonization of the plant host. This may facilitate the colonization process by evading the detection system of the hosts. The invader would lose virulence factors present on the surface of the cell wall that allows detection by the plant. This step would resemble the entrance of the arbuscule into the mycorrhizal plant cells that result in the formation of the specific interface between the partners that facilitate the transfer of substances between the partners (Brundrett, 2002). Interestingly, endophytic fungi lack this specialized interface and communicate with their hosts using relatively unspecialized hyphae (Brundrett, 2006).

In contrast to mycorrhizal fungi, endophytic fungi possess the ability to colonize plants belonging to the Brassicaceae, which are particularly abundant in metal hyper-accumulators and metallophytes; hyper-accumulators have been found in 45 families of plants with a large number belonging to this family (van der Ent et al., 2013). Similar to the mycorrhizal fungi, endophytic fungi were shown to promote plant growth under nutrient-limiting conditions (Hiruma et al., 2018). Such conditions are frequently encountered in metalliferous soils. In general, the Brassicaceae do not associate with mycorrhizae, probably due to the loss of symbiotic genes and the lowered expression of the nucleotide-binding site leucine-rich repeat resistance proteins (NLRs) required for the arbuscular mycorrhizal fungi (AMF) (reviewed in Hiruma et al., 2018). However, there were a few reports of AM fungi present in some Brassicaceae, such as the Cd hyper-accumulator *Biscutella laevigata* (Orłowska et al., 2002) or the Zn hyper-accumulator *Thlaspi caerulescens* (Regvar et al., 2003) collected from sites rich in TM. These cases are presently interpreted as the endophytic growth of mycorrhizal fungi, since the presence of arbuscules is mostly ephemeral, often only observed at the flowering stage, and the cases are rare, being observed only under field conditions and not confirmed under greenhouse conditions (Brundrett and Tedersoo, 2018). While endophytic fungi dominate non-mycorrhizal plants, mycorrhizal plants are also inhabited by numerous endophytes, including species of fungi that inhabit the Brassicaceae (Chen et al., 2012; Wężowicz et al., 2014; Sim et al., 2018). This finding is particularly interesting in the context of symbiotic fungi function. If the roles of these different symbionts overlap, how do they affect each other’s function? Another pending question is what are the specific functions of the endophyte that distinguish them from mycorrhiza? It appears that the response of the plants to inoculation with a single fungus (mycorrhizal or endophyte) differs from the response to inoculation with species of both groups of fungi (Wężowicz et al., 2017; Berthelot et al., 2018; Ważny et al., 2018; Zhou et al., 2018). The costs of harboring multiple fungal symbionts are higher but increased gains compensate for this (Field et al., 2016). The interaction between these two groups of fungi *in planta* or the effect of the presence of mycorrhizal fungi on the diversity and function of fungal endophytes has not been thoroughly investigated. Such studies would broaden our understanding of the role of different fungi in plant biology and the plasticity of the plant-fungus interaction. Available studies indicate that fungal endophytes may play a role similar to that of mycorrhizae (Rodriguez et al., 2009; Rudgers and Swafford, 2009; Andrade-Linares and Franken, 2013; Hiruma et al., 2018). How this role is affected by the presence of mycorrhizal fungi and how the fungal lifestyles change during the presence of a potential competitor remain to be investigated.

## Toxic Metal Tolerance of Endophytic Fungi

Certain strains of endophytic fungi exhibit extraordinarily high resistance to toxic metals. In culture, endophytic fungi can withstand TM concentrations in the mM ranges (Deng et al., 2011; Khan et al., 2017a; Domka et al., 2019). This adaptation provides a competitive edge over non-adapted fungi in colonizing plants in metalliferous habitats (Gadd, 2016). The role of symbiosis in the evolution of the fungal TM tolerance is unknown. It remains to be elucidated whether the TM tolerant plant served as shelter for the fungus, allowing it to evolve metal tolerance under the TM stress, or whether the metal-tolerant fungus facilitates the growth of the host plant in metalliferous soils. In the case of the former, the ability to colonize plant tissues in addition to its metal tolerance would play a role in determining the adaptation of the endophytic fungi to metal toxicity.

Many TM-tolerant fungal species, such as representatives of the genera *Phomopsis* and *Bipolaris* (DSE, class 4), have been isolated from many plant families (Ban et al., 2012; Wężowicz et al., 2014; Zhang et al., 2014; Sim et al., 2018; Domka et al., 2019). Studies indicate that endophytic fungi isolated from plants growing in areas polluted with TM are more tolerant to pollution and thus can be potentially more appealing for use in microbial-assisted phytoremediation. For example, DSEs isolated from *Alnus nepalensis* were shown to possess a higher tolerance to Cd than isolates from less polluted stands (Xu et al., 2015). In another study, 50% of the endophytic fungi isolated from six dominant plant species growing on Pb-Zn wastelands were shown to be tolerant to Pb^2+^ or Zn^2+^ (Li et al., 2012a). In addition, the growth of some of these fungi was even stimulated by the TM. The authors suggested that certain fungal species could adapt to metal toxicity due to their long-time exposure to the TM. Two strains of the endophytic fungi isolated from *Solanum nigrum* L. growing in a Cd-contaminated environment possessed the highest tolerance to Cd concentrations of up to 2 mM (Khan et al., 2017b). Similarly, Sim et al. (2018) isolated highly tolerant fungal endophytes species from *Phragmites* sp. utilized in wastewater cleanup. The majority of the fungi were able to withstand Pb, Zn, and Cu concentrations over 200 mg l^−1^. Interestingly, Gadd (2016) reported that certain fungi that are not necessarily fungal endophytes not adapted to TM can exhibit significant TM tolerance and become dominant in environments polluted with heavy metals; thus, resistance is not necessarily related to their environmental origin. The TM tolerance of some non-adapted fungi was shown to be similar to that of the fungal strains from the sites polluted with TM (Gadd, 2016). This finding suggests that inherent tolerance (probably not TM-related) mechanisms may have been the determining factor in the selection of the fungal endophytes that inhabit metalliferous environments.

High quantities of toxic metals deposited in soils cause qualitative and quantitative changes in microbial populations (Chodak et al., 2013; Corneo et al., 2013). Li et al. (2016) investigated the diversity of endophytic fungi isolated from *Dysphania ambrosioides* occurring at two TM-polluted stands. These researchers identified significant differences in the fungal population structure and dominant genera that were present between the sites analyzed. The colonization rate of the plants selected from stands highly polluted with TM was significantly lower than those from less polluted locations. Among all the strains isolated, regardless of origin, 50% were shown to be TM tolerant. Dominant genera exhibited higher tolerance. However, more metal tolerant isolates were found in plants from the less polluted stand. There were no differences in the Shannon index (H′) between the sites, which indicate similarity in fungal diversity. Interestingly, according to Wężowicz et al. (2014) fungal populations isolated from *Verbascum lychnitis* growing on post-mining tailings were more diverse than those from the non-polluted sites.

### Mechanisms of Fungal Metal Tolerance – The Role of Glutathione

Metal tolerance mechanisms in fungi that are described most frequently involve glutathione (GSH) and GSH-related tolerance that are induced in response to a variety of environmental factors (Na and Salt, 2011; Saraswat and Rai, 2011). Thus, the tolerant fungal strains, not necessarily TM adapted, that possessed the ability to colonize plants would be the driving force in the plant-fungal symbiosis in habitats polluted with heavy metals. Although endophytic fungi were shown to be able to accumulate extremely high quantities of TM in their mycelia, there is a lack of a comprehensive description of the mechanisms of metal transport, sequestration, and detoxification for this group of fungi (Deng et al., 2014; Zahoor et al., 2017). Zhao et al. (2015a) performed a transcriptome analysis of *Exophiala pisciphila* (DSE) growing in the presence of Cd using RNA-Seq. These researchers found several upregulated genes associated with TM binding, transport and detoxification, reactive oxygen species scavenging, redox homeostasis maintenance, and sulfur assimilation. The authors suggested that sulfate-containing molecules could increase during Cd stress, including glutathione (GSH), phytochelatins, and metallothioneins (Na and Salt, 2011). After uptake, the toxic metals are neutralized by complexation in the cytosol and translocated for storage in the vacuole. The vacuolar compartmentalization of metal chelates enables the fungi to isolate potentially dangerous pollutants from sensitive cellular compartments. Out of the numerous metal chelating molecules present in living organisms, the thiol-containing substances GSH (glutathione) and metallothioneins were shown to be present in, e.g., mycorrhizal fungi (Saraswat and Rai, 2011). The tripeptide GSH (γ-glu-cys-gly) is a non-protein thiol found abundantly in all cell compartments. In addition to its ability to sequester metals, it acts as a potent antioxidant and removes the damage caused by oxidative stress (Freeman, 2004). In addition, Ban et al. (2012, 2017) reported that Pb treatment leads to a transient GSH increase in non-pathogenic *Gaeumannomyces cylindrosporus* (DSE) that improves tolerance to Pb. Apart from GSH-dependent tolerance, Wei et al. (2016) analyzed the role of an *Nramp* (*natural resistance-associated macrophage protein*) gene from the DSE *E. pisciphila*. This gene encodes the plasma membrane bivalent cation transporter. The expression of *EpNramp* is downregulated after Cd exposure. Its heterologous expression in yeast Fe-uptake mutants leads to growth complementation. The authors suggested that this protein is partially involved in the tolerance of *E. pisciphila* to cadmium.

### Fungal Endophytes Limit Metal Toxicity by Exuding Metal-Binding Molecules Into the Rhizosphere

In addition to immobilizing the TM within its hyphae, fungi can limit the toxicity of TM by secreting various types of agents that chelate metals (Meharg, 2003; Bellion et al., 2006; Cabral et al., 2015), such as citrates or oxalic acid (Gadd, 1999). Available reports indicate that AMF are not able to secrete these types of organic acids but were shown to synthesize and release the glycoprotein glomalin (reviewed in Meharg, 2003; González-Chávez et al., 2004). Chelator exudation is an important mechanism of TM stabilization in the soil and prevents the entry of TM into the plant roots. The synthesis and secretion of metal-chelating molecules, including organic acids, siderophores, exopolysaccharides (EPSs), and phenolic compounds by fungal endophytes similarly to other mycorrhizal fungi with the exception of AMF, were reported (Bartholdy et al., 2001; Koulman et al., 2012; Zhao et al., 2015b; Garza et al., 2016; Gupta and Joia, 2016; Yamaji et al., 2016; Tong et al., 2017). In addition, a small number of reports indicate that fungal endophytes may bind TM ions to their cell wall components, similar to mycorrhizae (Tong et al., 2017). The production of siderophores by endophytic fungi was reported on a few occasions. Koulman et al. (2012) showed that the clavicipitaceous endophyte *Epichloë festucae* synthesizes siderophores with an unusual structure. In another study, Johnson et al. (2013) showed that the *E. festucae sid* gene, encoding a siderophore synthase, is necessary to establish symbiosis between the fungus and *Lolium perenne*. The plants infected with the Δ*sidN* mutants were stunted, and detailed microscopic studies revealed abnormalities in the distribution and localization of the mutant hyphae (Johnson et al., 2013). Bartholdy et al. (2001) reported the production of siderophores by the DSE fungus *Phialocephala fortinii*. HPLC analysis identified ferricrocin, ferrirubin, and ferrichrome C in the culture filtrate. Experiments performed by Yamaji et al. (2016) showed that the two DSE strains *P. fortninii* and *Rhizodermea veluwensis* were able to produce siderophores that are probably involved in the TM immobilization in the rhizosphere. Ban et al. (2012) reported that the melanin content in *G. cylindrosporus* increased following supplementation with Pb. Melanin is considered to be the most important component of the fungal cell wall involved in the alleviation of TM stress. There are numerous studies reporting its ability to bind metal ions; however, the role of melanin in the TM tolerance of the DSE remains unclear (Bruenger et al., 1967; Gadd, 1993; Fogarty and Tobin, 1996; Ban et al., 2012).

In addition, most endophytic fungi studied to date were shown to possess the ability to form nanoparticles (NPs), what may subsequently reduce the metal toxicity exerted on the host plants, as well as on the fungi (Durán et al., 2011). The efficiency of NPs mycosynthesis differed in between species of fungi (Shankar et al., 2003; Qian et al., 2013; Devi and Joshi, 2015). Recent studies indicate that NPs may be used in agriculture as nanofertilizers (Liu and Lal, 2015) that facilitate uptake of micronutrients (Dimkpa et al., 2015). Fungi utilize cellular enzymes, proteins, and membrane-bound molecules as electron donors in the reduction reaction. Reduced metal ions can be precipitated as NPs intracellularly or extracellularly (reviewed in El Enshasy et al., 2018). It was proposed that during intracellular synthesis metal ions are at first electrostatically bound to the fungal cell wall or diffuse through it and are subsequently reduced and precipitated by enzymes present in the cytoplasmic membrane (e.g., ATPases, hydrogenases) (Vahabi and Dorcheh, 2014). This leads to nanoparticle formation. The extracellular synthesis of nanoparticles occurs through the release of reductase enzymes (e.g., nitrate reductase, NADPH-dependent reductases, and FAD-dependent glutathione reductase) by metal exposed fungi and subsequent reduction of metal ions to form NPs (Kashyap et al., 2013; Singh et al., 2016; El Enshasy et al., 2018). Devi and Joshi (2015) reported the ability of three endophytic fungi to biosynthesize silver NPs. Similar properties were shown for the endophytic *Epicoccum nigrum* (DSE) and *Colletotrichum* sp. (Shankar et al., 2003; Qian et al., 2013). Also, Netala et al. (2016) have shown that the synthesis of silver NPs by the endophytic fungus *Pestalotiopsis microspora* VJ1/VS Yeasts from the *Cryptococcus* and *Rhodotorula* genus was also shown to produce silver NPs on several occasions (Salvadori et al., 2014; Fernández et al., 2016). Data concerning the ability of mycorrhizal fungi to synthesize NPs are scarce. Manceau et al. (2008) investigated copper speciation at the soil-root interface and found that copper was biotically reduced to NPs by mycorrhizal plants. Also, González-Chávez et al. (2002) reported the formation of electron-dense Cu granules within hyphae of AMF isolated from Cu- and As-contaminated soil, what suggests that mycorrhizal fungi also can produce nanoparticulate copper. However, more studies are required to understand the synthesis of the NPs in the plant rhizosphere, their effect on plants and microbial tolerance to TM, and their potential use in phytoremediation strategies.

## Plant Metal Tolerance Induced by the Fungi

**Figure 2:**
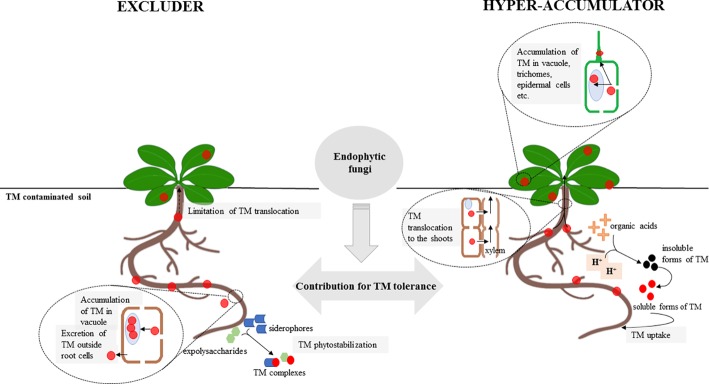
Comparison of the effect of endophytic fungi on toxic metal (TM) uptake and translocation in an excluder (left) and a hyper-accumulator (right) plant. Plants inhabiting sites rich in toxic metals may express two different strategies: TM avoidance/exclusion or hyper-accumulation. Endophytic fungi affect these pathways directly through the regulation of endogenous host plant mechanisms involved in TM tolerance or/and indirectly by the accumulation of TM in the mycelium, TM sorption into fungal cell walls, or the secretion of chelating agents, such as organic acids, phenolic compounds, and exopolysaccharides. Thus, the influence of endophytic fungi leads to the enhancement of the excluder/hyper-accumulator phenotype expressed as changes in the TM level in plant organs. See the text for more detail.

Plants inhabiting sites rich in toxic metals (TMs) developed a variety of mechanisms that allow them to survive in extremely adverse environments. Two primary strategies have evolved as an adaptation to metal toxicity. Plants may either accumulate high quantities of TM and internally neutralize toxic elements by a highly effective and complex detoxifying system or employ avoidance/exclusion strategies that limit metal toxicity (reviewed in DalCorso et al., 2013). According to available studies, fungal endophytes may impact both strategies depending on the strategy employed by its host (Jiang et al., 2008; Deng et al., 2013, 2014; Zhu et al., 2015; Zahoor et al., 2017; Rozpądek et al., 2018) (Figure 2) (Table 1). For example, Rozpądek et al. (2018) showed that endophytic *Mucor* sp. affects TM avoidance strategy of *Arabidopsis arenosa*, leading to the decrease of Zn and Fe uptake by the plant and simultaneously to upregulation of root to shoot translocation of Zn, Fe, and Cd. During interaction with the endophyte, plant genes implicated in metal homeostasis were affected. In turn, Jiang et al. (2008) revealed that *Brassica juncea* inoculated with Acacia-derived endophytic fungi was able to accumulate more Cd and Ni, depending on TM present in the soil, than wild type. These findings imply that the metal tolerance mechanisms of the host affected by the symbiotic fungal endophytes are not specific or novel but rather are differentially expressed and regulated. An important aspect of plant metal stress tolerance is the optimal metal distribution between the plant organs and the cell compartments that enable effective metal detoxification and protect the sensitive cellular compartments from metal toxicity. The accumulation of high quantities of TM has received a substantial amount of attention due to the importance of hyper-accumulating plants in phytoremediation and phytomining (reviewed in Verbruggen et al., 2009; van der Ent et al., 2013). Plant metallophytes have evolved highly effective metal detoxification mechanisms. In the case of hyper-accumulators, these mechanisms allow them to translocate and accumulate high quantities of metals in their shoots, which are associated with the upregulation of the genes encoding metal transporters enabling them to optimize metal distribution within the plant (Verbruggen et al., 2009). The number of studies describing the role of fungi, primarily AMF (Chen et al., 2003, 2004; Leung et al., 2006; Vogel-Mikuš et al., 2006; Punamiya et al., 2010), in the adaptation of hyper-accumulators is limited, and to our knowledge, the endophytic mycobiota of this group of plants has not been thoroughly investigated. In the work of Vogel-Mikuš et al. (2006), the influence of AM fungi on uptake of Zn, Cd, and Pb by *Thlaspi praecox* was investigated. The authors revealed that AM colonization resulted in higher nutrient uptake and lower TM accumulation by plants. In contrast, Punamiya et al. (2010) showed that *Chrysopogon zizanioides* accumulated more Pb in association with the AMF *Funneliformis mosseae.*

**Table 1:** Comparison of plant toxic metal tolerance mechanisms activated by symbiotic fungi.

Mechanism		Ectomycorrhizal fungi (ECM)	Arbuscular mycorrhizal fungi (AMF)	Endophytic fungi
TM avoidance	Reduced TM transfer to the plant	• Biosorption (Chalot et al., 2000; Colpaert et al., 2011) • Bioaccumulation (Turnau et al., 1996)	• Biosorption (Cabral et al., 2015) • Bioaccumulation (Janoušková et al., 2006; Meier et al., 2012; Corneo et al., 2013)	• Biosorption (Tong et al., 2017) • Bioaccumulation (Zahoor et al., 2017)
	Reduced TM bioavailability	• Metal transformation (Gadd, 1993) • Secretion of chelating agents (e.g., organic acids) (Bellion et al., 2006; Ray and Adholeya, 2009)	• Metal transformation (Gadd, 1993; Wu et al., 2015) • Secretion of chelating agents (e.g., organic acids, glomalin) (González-Chávez et al., 2004; Cabral et al., 2015)	• Metal transformation (Gadd, 1993) • Secretion of chelating agents (e.g., organic acids, siderophores, phenolic compounds) (Bartholdy et al., 2001; Koulman et al., 2012; Zhao et al., 2015b; Yamaji et al., 2016; Tong et al., 2017)
Increased TM concentration in the plant	Increased availability of TM	• Secretion of chelating agents (Fomina et al., 2005; Machuca et al., 2007; Vamerali et al., 2010) • Metal transformation (Gadd, 1993)	• Secretion of chelating agents (Göhre and Paszkowski, 2006) • Metal transformation (Gadd, 1993; Li et al., 2016)	• Metal transformation (Gadd, 1993)
	Increased TM uptake and translocation	• Indirectly through plant growth promotion (Sell et al., 2005) • Regulation of the expression of host genes encoding metal transporters (Ma et al., 2014)	• Metal transport from fungal hyphae to plant cells (Göhre and Paszkowski, 2006) • Indirectly through plant growth promotion (Usman and Mohamed, 2009) • Regulation of the expression of host genes encoding metal transporters (Burleigh et al., 2003; Bona et al., 2010)	• Indirectly through plant growth promotion (Zhu et al., 2015)

### An Extension/Filter of the Root?

Fungi can efficiently immobilize TM and limit metal uptake by the plant due to the large surface of the mycelia and the development of various mechanisms to tolerate and detoxify metals. This finding is particularly important because the hyphae of symbiotic fungi spread out from the root into the surrounding soil and significantly extend the root surface area. It was suggested that the extraradical hyphae of the AMF could represent up to 90% of the total AMF biomass and account for approximately 25% of the total microbial biomass in agricultural soils (Leake et al., 2004). This allows the efficient supply of the plant with the simultaneous filtering of the uptake of nutrients and water (Joner, 2000). Arbuscular mycorrhizal symbiosis was shown to significantly alter the root morphology, usually leading to a reduction in the root/shoot ratio. These changes result in a high degree of dependence of the plant on the symbiosis; AMF are responsible for the majority of nutrient absorption and delivery to the host plant (Sanders et al., 1977; Hetrick, 1991). In ectomycorrhizal roots (ECM), as an adaptation to symbiosis, the number and length of the root hairs are significantly limited. This finding implies that as in AMF, the majority of water and nutrient acquisition is transmitted through the fungal hyphae (Hetrick, 1991). Turnau et al. (1996) revealed the filtering role of the ectomycorrhizal fungus *Rhizopogon roseolus* hyphal mantle formed on *Pinus sylvestris* roots. The authors investigated the distribution of potentially toxic metals within mycorrhizae and showed the enhanced accumulation of Cd and Al in the fungal mantle and their gradual decrease along the Hartig net toward the inside of the root. A similar filtering effect was observed in the *Suillus luteus/P. sylvestris* mycorrhizae in which Ca, Fe, Zn, and Pb were concentrated in the mantle and rhizomorph (Turnau et al., 2001). A limited number of studies indicate that some species of root-bound endophytic fungi (e.g., *Colletotrichum tofieldiae, Serendipita indica,* Helotiales F229) may behave similarly to mycorrhizae and extend out of the root to increase the root surface area and transmit nutrients to the plant (Yadav et al., 2010; Hiruma et al., 2016; Almario et al., 2017). However, this phenomenon has not been confirmed in the presence of TM. The fungi’s role as a physical barrier preventing contact with TM cannot be ruled out, but to the best of our knowledge, there are no studies that would provide clear evidence that endophytic fungi entwine plant roots and serve as a physical protection against TM. Additionally, several distantly related groups of endophytic fungi were shown to activate root hair elongation and induce other alterations in the root architecture leading to an increase in the root surface (Harman et al., 2004; Stewart and Hill, 2014; Hiruma et al., 2016). One type of fungus, *Mucor* sp., was shown to activate the root hair elongation of its host *A. arenosa* in non-polluted soil, as well as in substrates polluted with TM (Rozpądek et al., 2018). In mycorrhizal plants, the fungal hyphae partly take over the root functions, enabling the plant to grow relatively smaller roots that are less specialized for water and nutrient uptake. Endophytic fungi activate root growth and thus allow the plant to penetrate the surrounding soil more thoroughly but with its own tissues. It cannot be ruled out that fungal endophytes increase the surface of the plant root and simultaneously extend into the rhizosphere and serve as a filter that binds and prevents TM uptake by the plant. This situation may be true in the case of non-mycorrhizal plants, but in nature, most plants are inhabited by endophytic fungi and other fungal symbionts, including mycorrhizae (Wężowicz et al., 2017). Fungal endophytes were shown to occur within the roots and form complex webs with mycorrhizal hyphae (Horton et al., 1998; Agler et al., 2016). The majority of the studies available describe plant-fungal interactions in single inoculation experiments. This approach has several advantages but unfortunately neglects the full spectrum of the plant-fungus and symbiotic fungus-fungus interactions that occur in natural environments. The plant response to symbiotic microorganisms appears to be far more complex than the responses described in single inoculation experiments, and its description needs to include the complexity of microorganisms inhabiting its host.

### Endophytic Fungi Affect Toxic Metal Uptake and Distribution Within the Plant

After overcoming the physical barriers of the root, the TM efflux into the cell, vacuolar sequestration, and xylem loading is coordinated by a number of heavy metal transporters, a broad group of different proteins, such as CPx-ATPases for Cu or Cd, ABC transporters for Cd transport into the vacuole, ZIP transporters (ZRT- and IRT-related proteins for Fe or Zn, respectively), and Nramp transporters. Although this network is thought to balance the concentration and partitioning of essential metals, such as Zn, it also unselectively transports toxic elements, such as Cd (reviewed in DalCorso et al., 2013). Metal-specific and non-specific transporter proteins enable metal compartmentalization and subsequent stress alleviation by toxic metal complexation with organic molecules, such as metallothioneins, organic acids, and phytochelatins (PCs) that are synthesized by the plant. The abundance of these molecules based on studies with overexpression lines and hyper-accumulators correlates with the ability to accumulate TM (Freeman, 2004; Janoušková et al., 2005).

There are several studies that confirm the influence of endophytic fungi on the TM uptake and distribution in the host plants. Inoculation with the fungal endophyte *Mucor* sp. (class 2) isolated from plants growing on soils heavily polluted with TM resulted in a decrease in Cr uptake of up to 90% by *Brassica campestris.* The fungus was able to bio-transform the metals deposited in the soil and accumulate them in its hyphae, thereby making them less available to the plant (Zahoor et al., 2017). Recently, similar results were obtained for a strain of *Mucor* sp. isolated from *A. arenosa* inhibiting the “Bolesław” Zn, Cd, and Pb mine dump in southern Poland. Inoculation with the fungal strain UNIJAG.PL50 improved the production of plant biomass, decreased the accumulation of metals, and significantly affected the metal distribution within the plant, leading to more effective TM translocation to the above-ground parts and subsequently to more uniform TM distribution (Rozpądek et al., 2018). The same *Mucor* sp. strain was simultaneously used with *Rhizoglomus intraradices* and induced increased Zn uptake in *Lactuca serriola* growing on TM-rich industrial wastes. The concentration of Zn was higher in both the roots and shoots of the symbiotic plants (Ważny et al., 2018). In turn, the inoculation of maize with the TM-tolerant DSE *E. pisciphila* strain H93 alleviated the deleterious effects of the presence of TM by reducing the translocation of the HM ions from the roots to the shoots (Li et al., 2011). Similarly, He et al. (2017) reported the positive effect of *E. pisciphila* on maize growth under high Cd and the reduced translocation of the Cd to the shoots. Yamaji et al. (2016) showed that *Clethra barbinervis* inoculated with its fungal endophytes accumulated less TM than the control plants, while Zhu et al. (2015) reported that two species belonging to the *Mucor* genus, *M. circinelloides* (Z4) and *M. racemosus* (Z8), improved the growth and the accumulation of Pb and Cd of Guizhou oilseed rape. Both strains increased the accumulation of the TM in the plant by 117.6% for Cd and 63.48% for Pb, simultaneously reducing their concentrations in the substratum by 60.57% and 27.12%, respectively. Another endophyte that was shown to improve the growth of *Brassica napus* and *B. campestris* in TM-polluted soil was *Rhodotorula* sp. This group of yeast endophytes (class 2) is particularly interesting due to their ability to produce exopolysaccharides (EPSs) with a high potential to absorb pollutants, including TM (Garza et al., 2016; Fernández et al., 2018). Inoculation with this *Rhodotorula* sp. increased the efficiency of the plant to extract Cd, Cu, and Pb (Wang et al., 2013). In addition, Deng et al. (2014) demonstrated that *Lasiodiplodia* sp. was an endophytic fungus of rape that showed promise for bioremediation. The fungal strain increases the translocation rate of the Cd from the roots to the shoots. *Solanum nigrum* was shown to accumulate more Cd in the roots and shoots after inoculation with the DSE *Phomopsis fukushii* strain PDL-10. He et al. (2017) showed similar results. Studies reported the influence of *E. pisciphila* on maize root-colonizing DSE and Cd tolerance. Plants inoculated with the fungus accumulated more Cd in the roots, and the metal translocation to the above-ground parts was limited (He et al., 2017). Ni uptake and most importantly extraction efficiency were positively affected in *Brassica juncea* inoculated with *Trichoderma atroviride* strain F6 (also belonging to class 4, root inhabiting Hypocreales). In addition, the inoculated *B. juncea* exhibited a higher tolerance to metal toxicity (Cao et al., 2008).

Despite the increasing number of reports examining the influence of endophytic fungi on TM uptake and translocation in the host plant, only a small number of studies attempted to investigate the role of endophytic fungi with respect to the mechanism of this aspect of plant TM metabolism. Rozpądek et al. (2018) showed that the reduction of metal accumulation and changes in their distribution within *A. arenosa* were accompanied by the upregulation of several metal homeostasis-related genes involved in metal exclusion, sequestration in the vacuole, and ROS (reactive oxygen species) scavenging. The upregulated genes include *hma3 (heavy metal associated 3), mtp1 (metal transport protein 1), zif1 (zinc induced facilitator 1),* and *cax2* (*cation exchanger 2*) that encode tonoplast-bound vacuole carrier proteins (Rozpądek et al., 2018). The overexpression or complementation with these genes conferred tolerance to metal stress to plants and yeast, respectively (Haydon and Cobbett, 2007; Koren’kov et al., 2007; Morel et al., 2009). In hyper-accumulating *T. caerulescens* and *Arabidopsis halleri,* phloem loading is accomplished by the highly expressed *hma2* (*heavy metal associated 2*) and *hma4* (*heavy metal associated 4*). To date, there have been no reports indicating that fungal endophytes may affect metal translocation from the root to the shoot by interfering with the expression of these metal transporters. However, the expression of *pcr2* (*plant cadmium resistance 2*) involved in Zn phloem loading and metal efflux out of the root epidermal cells (Song et al., 2010) was upregulated, and metal accumulation was higher in the shoots of inoculated plants (Rozpądek et al., 2018). In another study, Wang et al. (2016) analyzed the response of maize inoculated with DSE in the presence of Cd. These researchers showed that inoculation with the endophytes led to the inhibition of an unspecified *zip* gene, which coincided with the decreased uptake of Cd following DSE infection. In addition, *mpt1* expression was also activated in the plants inoculated with DSE during Cd toxicity.

Mycorrhizal fungi were also shown to affect the uptake of TM and their subsequent translocation and accumulation by the host plants (Table 1). The expression of metal transporter-encoding genes, including proteins belonging to Nramp, Zip families, HMA4 transporter, or arsenic putative transporter POR 29, was activated in the AMF plants (Burleigh et al., 2003; Ouziad et al., 2005; Bona et al., 2010; Ma et al., 2014). Based on the current knowledge, the AM fungi, depending on the fungal and plant species, may inhibit the TM uptake by the host plant or increase the metal translocation to the above-ground parts of the plant (Joner and Leyval, 1997; Chen et al., 2007). Ectomycorrhizal fungi were proven to serve as a barrier against TM uptake, since they were reported to inhibit the uptake, translocation, and accumulation of the TM in the upper parts of the plant (Adriaensen et al., 2004, 2005, 2006; Walker et al., 2004). Depending on the fungal species, the TMs were shown to accumulate mostly in the hyphal mantle or in the extramatrical mycelia (Turnau et al., 1996, 2002).

### The Influence of Endophytic Fungi on TM Detoxification by the Plant

Endophytic fungi were also shown to affect the expression of the stress-related genes of the host plant, thus triggering resistance against TM toxicity. In maize, inoculation with the DSE *E. pisciphila* (Wang et al., 2016) led to the upregulation of *pcs* expression. The authors suggested that this could facilitate the transpeptidation of glutathione, as well as other thiols. Khan and Lee (2013) reported that soybean plants inoculated with the class 3 endophytic *Penicillium funiculosum* produced more reduced glutathione during Cu stress than control non-inoculated plants. In another study by the same authors, the interaction of the plant with endophytic strain RSF-6L led to increased catalase activity in *S. nigrum* during Cd toxicity (Khan et al., 2017a). The effect of inoculation on the activity and abundance of antioxidants was confirmed in several other studies (Wang et al., 2016). Endophytic *Trichoderma atroviride* and *T. virens* (class 4) were shown to influence the levels of expression of the *adc1* and *adc2* genes implicated in spermidine biosynthesis in *A. thaliana* in the control (Salazar-Badillo et al., 2015). The synthesis of the stress protective polyamines spermidine and spermine is often activated following exposure to TM, and their abundance often correlates with stress tolerance. However, there is a lack of studies indicating the role of polyamines in the resistance to TM triggered by the fungal endophytes.

Gene expression studies confirm the role of mycorrhizae in conferring plant tolerance against TM by regulating the expression of the plant stress response genes. The genes that encode plant defense compounds that are described the most frequently are those involved in the upregulation of sulfur metabolism (sulfur containing compounds), such as metallothionein, GSH-synthase, ascorbate peroxidase, or glutathione S-transferase (Bona et al., 2010; Cicatelli et al., 2012; Lingua et al., 2012; Shahabivand et al., 2017).

### Plant Growth Promotion Imposed by the Fungi

There is no unequivocal “mode” of fungal action in terms of the effect on metal uptake and distribution. Some species of fungi protect the plant by decreasing metal uptake, while other species increase uptake and translocation from the root to the shoot. In addition, there is no consensus regarding the mechanism of the alleviation of metal toxicity by fungi. It remains to be elucidated whether symbiotic fungi improve plant growth during metal toxicity by facilitating water and nutrient uptake by the plant and thus indirectly by fine tuning the metal tolerance of the host or directly by upregulating specific mechanisms of metal tolerance. In the majority of studies available, the plants inoculated with endophytic fungi produce more biomass during metal toxicity than the non-inoculated plants (Zhu et al., 2015; Wang et al., 2016; Rozpądek et al., 2018). However, it is not clear whether this is a result of fungal dependent stress alleviation or a strategy that allows the plants to “dilute” the accumulated metals due to improved growth as a result of enhanced nutrition. Interestingly, the ability to facilitate nutrient uptake was a trait that distinguished the fungal endophytes from the mycorrhizae. Endophytes were thought to be unable to improve nutrient uptake by the plant, but in recent years, fungal endophytes were shown to participate in plant nutrition (Brundrett et al., 2002). Fungal endophytes improve plant water and nutrient uptake. It has been reported that toxic metals often interfere with the root uptake of nutrients, such as Fe, P, Mg, K, Ca, and Zn, and with the metabolic functions of the essential nutrients, leading to plant growth retardation (Ouzounidou et al., 2006). In addition, soils augmented with toxic metals are often highly deficient in nutrients, which impose additional restraints to plant growth. Under such conditions, the fungal endophytes improve the acquisition of plant nutrients by mobilizing nutrients and making them available to the plant roots (Cui and Nobel, 2006; Weyens et al., 2009a; Rajkumar et al., 2012). The Glomeromycota are obligatory biotrophs and lack saprotrophic capabilities. Thus, their ability to decompose organic matter and to liberate compounds that are absorbable for the plant into the soil is limited. In contrast, endophytic fungi are saprotrophic microorganisms that can improve plant nutrition by decomposing organic matter (Promputtha et al., 2007; Purahong and Hyde, 2011). This trait can be particularly useful in environments polluted with TMs that are devoid of available nutrients.

AMF develop specialized structures – arbuscules – that allow an exchange between the fungus and the plant. Abuscules are branched intracellular hyphae that are present in the root cortex. The absence of these structures in fungal endophytes suggests that the mechanism of nutrient transfer at the plant-endophyte interface differs from that employed by the AMF. Interestingly, the Mucoromycotina of the bryophytes formed characteristic intracellular coils of an unknown function (Field et al., 2015). We can only hypothesize that these structures may play a similar role in Mucoromycotina to that of the arbuscules in the AMF. Another possible way to improve plant nutrition by fungal endophytes is the rhizophagy cycle hypothesized to act in the interaction between plants and endophytic bacteria and yeast (Paungfoo-Lonhienne et al., 2010; White et al., 2018). The rhizophagy theory suggests that fungi and endophytic bacteria can be the prey of the plant root cells and serve as nourishing factors that enable the plants to survive even without available nutrients in the surroundings.

Endophytic fungi are associated with a wide range of plant growth promoting activities, including P solubilization and the production of siderophores and phytohormones (Chhabra and Dowling, 2017). The role of fungal endophytes in the delivery of P to the plant was described in numerous studies. Hiruma et al. (2016) showed that *C. tofieldiae* transfers P to the host plant and that the phosphate starvation response (PSR) mechanism that is active in plant tissues is responsible for the control of root colonization. In another study, Rozpądek et al. (2018) showed that *Mucor* sp. improved host plant nutrition by inducing the PSR and probably stimulating the plant to produce enzymes implicated in phosphorus acquisition. Yadav et al. (2010) reported that maize inoculated with *S. indica* accumulates higher levels of P than control plants. In addition, plants inoculated with *S. indica* with knocked down *PiPT* accumulated less P than plants inoculated with a wild-type fungus. This indicates that PiPT is involved in phosphorus transport and that *S. indica* improves the nutrition of the host plant. Improved host phosphorus acquisition by *S. indica* was conferred by Wu et al. (2018). The class 2 endophytic *Fusarium tricinctum* strain isolated from *S. nigrum* was shown to produce IAA and stimulate the growth of the shoots and roots during Cd toxicity (Khan et al., 2017a). Yamaji et al. (2016) reported that *Clethra barbinervis*, naturally occurring at mine sites, could tolerate high TM concentrations due to the support of DSE, including *P. fortinii*, *Rhizodermea veluwensis,* and *Rhizoscyphus* sp. Inoculation tests revealed that these fungal endophytes enhance plant growth by promoting the uptake of K.

## Hyper-Accumulators and Symbiotic Fungi

The role of microorganisms in the phenomenon of hyper-accumulation remains unknown, even though much progress in this matter has recently been accomplished (reviewed in Furini et al., 2015). Only 10% of the metal hyper-accumulating species have been examined for their ability to interact with rhizospheric microorganisms, mostly for their associations with bacteria (Li et al., 2007; Pal et al., 2007; Kidd et al., 2018).

Turnau and Mesjasz-Przybylowicz (2003) reported several Ni hyper-accumulating plants from ultramafic soils in South Africa that were abundantly colonized by AMF. Orłowska et al. (2013) reported that the Ni hyper-accumulating perennial *Berkheya coddii* inoculated with mycorrhizal fungi accumulated P, K, Mn, and Zn in the cortical layer of the lateral roots and in the vascular stele more strongly, while Ni was detected particularly in the vascular tissue in non-inoculated plants. The inoculated plants translocated Ni to the above-ground parts of the plant more efficiently. The effect of the endophytic fungi on these plants that are strongly dependent on mycorrhizae still awaits investigation, which could be useful for phytoextraction.

In recent years, there has been an increase in studies on the influence of endophytic fungi on hyper-accumulating plant species. Khan et al. (2017b) characterized the endophytic fungal community associated with Cd-hyper-accumulating *S. nigrum*. They isolated 42 culturable fungal strains that belonged to the Ascomycota. In another study, Khan et al. (2017a) tested endophytic fungal strains isolated from *S. nigrum* for Cd tolerance and accumulation potential. They showed that one of the strains isolated RSF-6L exhibited the ability to reduce Cd uptake in the plant. Both the translocation factor (TF) and the bio-concentration factor were lower in the plants inoculated with endophytes than the control (Khan et al., 2017a). Interestingly, earlier studies showed that inoculation with the mycorrhizal fungi *Claroideoglomus claroideum* and *R. intraradices* enhanced the accumulation of Zn in the plant tissues with the primary reservoir of Zn in the shoots (Marques et al., 2006). It would be interesting to compare these results with those of double inoculation and various metals. In addition, inoculation with the fungi conferred protection to the host plant, leading to an improvement in tolerance.

## Conclusions

The pollution of soils with toxic metals not only affects the composition and diversity of microbial communities, leading to a reduction in the overall abundance of microbial species, but also results in the enrichment of metal-tolerant microbial strains (Yao et al., 2003; Stefanowicz et al., 2008; Azarbad et al., 2013; Chen et al., 2014). These strains are extremely tolerant to metal toxicity and based on the studies available may facilitate plant growth in environments polluted with metals. The role of mycorrhizae in the adaptation of plants to metal pollution has been extensively studied over the last three decades. However, the role of other microbial components of the mycorrhizosphere cannot be overlooked. This is particularly important due to the fact that plants in their natural environments simultaneously interact with a wide array of microorganisms. In case of the Brassicaceae metallophytes and hyper-accumulators that lost the ability to interact with mycorrhizae fungal endophytes may play a role resembling mycorrhizae in the adaptation of non-mycorrhizal plants to metalliferous soils; however, the mechanisms of the action of endophytes may distinguish them from other symbiotic fungi (Table 1). In nature plants, both mycorrhizal and non-mycorrhizal and microorganisms form complex assemblages termed the holobiont. The functioning of this super organism is determined by relationships between the interacting organisms, thus understanding the complexity and routs of mutual communication will allow us to utilize the potential of plant-based clean-up technologies more effectively. In general, the influence of toxic metals on fungi and plants encompasses induced changes on the level of the epigenome, transcriptome, proteome, metabolome, and secretome. In addition, these changes further influence the interactions between the plant and symbiotic fungi and other soil microorganisms (Figure 1). Endophytic fungi may alleviate TM stress in the host plants in various manners. They can serve as a barrier and prevent the entry of metal ions into plant tissues and optimize metal distribution within the plant. These roles of fungi have been described on several occasions; however, very little is known about how the symbiont induces the metal phenotype in plants. One of the most pending questions is whether the metal adapted fungus directly affects metal specific tolerance mechanisms or is improved adaptation to metal toxicity a result of indirect action such as improving water and nutrient availability in the soil and their subsequent uptake by plants that confer plant fitness? Another question is how much the endophyte influence on the plant TM tolerance differs from the analogous impact of mycorrhizal fungi? To answer these questions more studies concerning the role of fungal symbionts in plant response to TM are needed. Bioremediation is a dynamically growing area of green biotechnology. Endophytes may have considerable potential applications not only in microbial-assisted phytoremediation practice but also in mycoremediation of the areas that cannot be used for plant growth.

## Author Contributions

AD, PR, and KT wrote the manuscript. AD and KT prepared the figures. AD and PR contributed equally to the manuscript.

### Conflict of Interest Statement

The authors declare that the research was conducted in the absence of any commercial or financial relationships that could be construed as a potential conflict of interest.
